# Optimizing TREC‐ and KREC‐based newborn screening: Risk‐stratified algorithms significantly reduce referrals

**DOI:** 10.1111/pai.70412

**Published:** 2026-07-08

**Authors:** Maarja Soomann, Seraina Prader, Susanna Sluka, Jana Pachlopnik Schmid, Johannes Trück

**Affiliations:** ^1^ Division of Immunology and The Children's Research Center University Children's Hospital Zurich, University of Zurich Zurich Switzerland; ^2^ Swiss Newborn Screening Laboratory University Children's Hospital Zurich Zurich Switzerland

**Keywords:** agammaglobulinemia, KREC, NBS, NBS algorithms, newborn screening, SCID, TREC

## Abstract

**Background:**

Newborn screening (NBS) quantifying T‐cell receptor excision circles with or without kappa‐deleting recombination excision circles (TREC, KREC) enables early detection of severe T‐ and/or B‐cell lymphopenia. However, both markers have limited specificity, often resulting in unnecessary referrals. We compared the effects of alternative risk‐stratification strategies to reduce referrals while avoiding missed cases and unnecessary delays.

**Methods:**

We modeled the effects of multiple TREC and KREC‐based NBS algorithms using a comprehensive real‐life dataset from the first 6 years of the Swiss NBS program. Stratification approaches included adjustment of cut‐offs and integration of clinical data such as gestational age (GA), postmenstrual age (PMA), inpatient vs. outpatient status, family history, and maternal immunosuppression.

**Results:**

Lowering cut‐offs alone reduced abnormal results by 42% for TREC and 64% for KREC. The most efficient TREC algorithm, based on exact TREC levels and post‐menstrual age in preterm infants, reduced referrals by 61% (*p* < .0001) but missed nearly half of non‐SCID T‐cell lymphopenias and delayed referral in 2/3 of outpatients who eventually required immunological evaluation. Integration of family history and clinical signs mitigated delays in some cases. For KREC, combining information on gestational age, maternal immunosuppression, and inpatient status enabled a >10‐fold reduction in referrals (*p* < .0001) while still identifying all confirmed agammaglobulinemia cases except two with λ5 deficiency.

**Conclusion:**

Risk‐stratified, multistep NBS algorithms incorporating readily available clinical data can substantially reduce unnecessary referrals while preserving detection of target conditions. For KREC, simple algorithmic adjustments allow marked improvement in specificity with minimal diagnostic loss.

**Key message**

Unnecessary referrals can be successfully reduced by applying multilayered algorithms stratifying risk based on factors associated with transiently abnormal findings (prematurity, inpatient vs. outpatient status, exposure to maternal immunosuppression in utero) and exact TREC results.

## 1 INTRODUCTION

Newborn screening (NBS) using quantification of T‐cell receptor excision circles (TREC) from dried blood spots (DBS)^1^ revolutionized the early detection of severe combined immunodeficiency (SCID), enabling timely interventions and improving survival.^2^, ^3^, ^4^ After TREC‐based assays were demonstrated to be robust in large‐scale NBS programs,^5^ combined real‐time polymerase chain reactions (PCR)‐based assays incorporating the quantification of kappa‐deleting recombination excision circles (KREC) – the B‐cell counterpart of TREC – were developed.^6^, ^7^ Although early small scale studies rarely identified agammaglobulinemia,^8^, ^9^, ^10^, ^11^, ^12^ several cases have since been reported.^12^, ^13^, ^14^, ^15^ However, both markers are not disease‐specific: they measure the output of early lymphocyte development^1^, ^6^, ^16^ and can be abnormal in various inborn errors of immunity (IEI), secondary immunodeficiencies, or even in healthy newborns due to transient conditions.^2^, ^15^, ^17^, ^18^, ^19^, ^20^, ^21^ While early detection of non‐SCID, non‐agammaglobulinemia lymphopenias may still be beneficial in selected cases,^20^, ^22^, ^23^, ^24^ most newborns with transient findings or false‐positive results require no intervention but experience unnecessary referrals and associated stress and parental anxiety. Balancing sensitivity with reduction of non‐actionable findings is a central challenge in population‐based screening.

Several strategies have been proposed to improve the specificity of TREC‐based programs, including lowering cut‐offs, repeat sampling, and second‐tier testing.^5^, ^8^, ^17^, ^18^, ^25^, ^26^, ^27^, ^28^, ^29^ International experiences vary: in Norway, integrating rapid second‐tier genetic testing reduced referrals but their approach requires rapid access to high‐throughput sequencing capacity and may miss genetically undefined SCID.^30^ In the Netherlands, using a different set of PCR primers in the TREC assay or epigenetic immune cell counting for second‐tier testing, as well as lowering cut‐offs and adjusting the screening algorithm, were evaluated. Overall, lowering TREC cut‐offs and requesting a second DBS for low but measurable values reduced referrals, though other risk‐stratification approaches were not explored.^17^ For KREC, no systematic evaluation of referral‐reduction strategies has been published.

In Switzerland, nationwide combined TREC and KREC‐based screening was implemented in 2019. With the accumulation of several years of program data, it is now possible to evaluate alternative algorithms tailored to local conditions. We aimed to model the impact of lowering cut‐offs and applying multistep, risk‐stratified algorithms for both TREC and KREC. Stratification was based on readily available or easily obtainable variables, including gestational and postmenstrual age (GA and PMA), inpatient status, family history, and maternal immunosuppression, with the goal of reducing unnecessary referrals while maintaining detection of clinically relevant cases.

## 2 METHODS

We conducted a retrospective modeling study using real‐life data from the Swiss NBS program. Combined TREC and KREC‐based screening was implemented nationwide on January 1, 2019. From January 2019 to February 2024, DBS were analyzed using the ImmunoIVD SPOT‐it™ TREC & KREC Screening Kit.^31^ Following the inclusion of spinal muscular atrophy (SMA) in the national screening panel on March 1, 2024, the ImmunoIVD SPOT‐it™ TREC, KREC & SMN1 Screening Kit was adopted. Between January 2019 and December 2020, screening cut‐offs were set at TREC <10 and KREC <6 copies/punch. From January 2021 to February 2024, these thresholds were reduced to TREC <6 and KREC <4 copies/punch in line with manufacturer recommendations. From March 2024 onwards, cut‐offs were adjusted to TREC <10 and KREC <4 copies/punch based on an internal validation study.

All abnormal results were reported to the Division of Immunology, University Children's Hospital Zurich, and prospectively registered in a central database alongside clinical details and follow‐up outcomes. Subsequent steps, including obtaining a repeat card or referring the newborn for immunological investigations, were determined by the clinicians of the Division of Immunology. The initial screening algorithm and confirmatory testing protocol have been described previously.^32^ In short, newborns with TREC 0–1 were directly referred; in those with TREC >1 or isolated abnormal KREC, a new DBS sample was obtained. The latter were only referred if the abnormal TREC or KREC findings persisted in the new sample. This algorithm was occasionally modified based on clinical judgment or specific circumstances, and was also adapted to permit multiple new DBS samples for hospitalized newborns and for cases with abnormal KREC results with a plausible secondary cause. In addition, for quality control purposes, a DBS sample was whenever possible obtained alongside confirmatory testing in referred newborns. For the present analysis, we included cases detected between January 1, 2019, and December 31, 2024.

Due to intrinsic differences in the urgency of further investigations in situations when a severe T‐cell versus a severe B‐cell deficiency is suspected, and to ensure comparability with other studies, “abnormal TREC” refers to newborns with TREC values below the program cut‐off regardless of KREC status, and “abnormal KREC” refers to newborns with KREC values below the program cut‐off but normal TREC values. A “referral” was defined as performing lymphocyte immunophenotyping, whether in an outpatient clinic or during an existing inpatient stay. “Moderate T‐cell lymphocytopenia (TCL)” denotes non‐SCID, non‐congenital athymia TCL without a clearly identifiable secondary cause, irrespective of B‐cell counts or whether a genetic diagnosis was established. In the TCL group, all newborns who had been put on at least *Pneumocystis jirovecii* pneumonia prophylaxis due to low CD4^+^ T‐cell counts (<1.0 × 10^9^/L) were considered to have “actionable” TCL; the remaining were considered “non‐actionable”. Among patients with B‐cell deficiencies, all children with a genetically confirmed IEI and advised to receive immunoglobulin substitution were considered “actionable”. “Transient finding” refers to an abnormal TREC or KREC result with normal subsequent testing and no evidence of persistent immunodeficiency.

The analysis had two parts. First, we evaluated the effect of lowering cut‐offs using data from the first 2 years of screening, when higher thresholds (TREC <10; KREC <6) were in place. Second, we developed and modeled alternative stratified algorithms separately for TREC and KREC. These algorithms were derived from published strategies^8^, ^12^, ^14^, ^17^, ^18^, ^25^, ^27^, ^33^, ^34^ and from our own program's experience, incorporating readily available or easily obtainable clinical variables such as exact TREC or KREC values (categorized as “urgent” for TREC <2 vs. “non‐urgent” for all others), GA at birth and PMA at sampling, inpatient vs. outpatient status, family history of SCID or agammaglobulinemia, clinical signs suggestive of Omenn's syndrome or syndromes associated with congenital athymia, and maternal immunosuppression during pregnancy. Newborns were excluded from the second part if results of a second DBS were unavailable or if their initial results were in intermediate ranges (TREC 6–9 or KREC 4–5 copies/punch), as these cases could not have been assigned consistently across algorithms.

All statistical analyses were conducted in R version 4.2.2^35^ using the packages listed in Table S1. Categorical variables are presented as counts and percentages and were compared using chi‐square or Fisher's exact test. Receiver operating characteristic (ROC) analysis was used for evaluation the impact of reducing cut‐offs. For each algorithm, we calculated numbers of referred and missed cases, as well as delayed diagnoses. We compared referred cases pairwise between different models. A *p*‐value <0.05 was considered statistically significant.

The study was approved by the Cantonal Ethics Commission of Zurich (2022‐01029). Written informed consent was obtained from parents or legal guardians of all newborns with abnormal screening results in accordance with the approved protocol.

## 3 RESULTS

### 3.1 Study population

Of the 516,494 screened newborns, 443 (0.086%) showed abnormal results in the first DBS sample. After excluding those without consent, 414 newborns (93.5%) were included in the analysis: 192 (46%) in the abnormal TREC group and 222 (54%) in the abnormal KREC group. The GA distribution among patients with a final diagnosis of an IEI ranged from 27 weeks in the most premature infant to term, with detailed distribution shown in Figure S1.

In the abnormal TREC group, 82 of 192 newborns (43%) underwent referral. An IEI was diagnosed in 56 of the 82 referred newborns (68%): 11 with SCID, 2 with congenital athymia, and 43 with moderate TCL (Table S2). Seven of the 82 referred newborns (9%) had reduced T‐cell counts due to reversible conditions,^15^ while 19 of these 82 (23%) had normal immunophenotyping (false positives). Repeat DBS testing led to normalization of TREC values in 73 of the 192 newborns with abnormal TREC (38%), eliminating the need for referral. A further 37 of the 192 with abnormal TREC (19%) remained inconclusive because 29 died before follow‐up testing could be completed and 8 were lost to follow‐up.

Among the 222 newborns in the abnormal KREC group, 26 (12%) newborns were referred. An IEI was diagnosed in 12 of these 26 referred newborns (46%): agammaglobulinemia in 11 infants and ataxia telangiectasia in 1. Eleven of the 26 referred newborns (42%) had transiently reduced B‐cells that normalized over time: linked to in utero exposure to B‐cell depleting therapies in 5,^36^ azathioprine in 2, and prematurity in 1; no clear cause was identified in the remaining 3. Three of the referred 26 newborns (12%) had normal initial B‐cell counts (false positives). In 188 of the 222 newborns with abnormal KREC (85%), KREC levels normalized on repeat DBS, avoiding referral. Eight of the 222 cases with abnormal KREC (4%) were inconclusive: 1 infant died before further evaluation and 7 were lost to follow‐up. After adjusting for excluded cases due to missing consent, the total estimated referral rate across the program was 0.022%.

### 3.2 Effect of decreasing cut‐offs

The effect of adjusting cut‐offs was analyzed in the subset of newborns screened during the first 2 years of the program, when the higher cut‐offs (TREC <10 and KREC <6 were in use): 116 newborns with abnormal TREC and 121 with abnormal KREC. Lowering the TREC threshold from <10 to <6 copies/punch reduced the number of abnormal results from 116 to 67 (a 42% reduction, *p* = .0004). The model predicted that no cases of SCID or congenital athymia would have been missed; however, six cases of moderate TCL would not have been detected, including three with 22q11.2 deletion syndrome, one with ataxia telangiectasia, one idiopathic case, and one case of unclear etiology without genetic testing (Figure S2), all actionable. According to the ROC analysis, using <6 as a cut‐off yielded 74% sensitivity compared to <10 whilst improving specificity (Figure S3A). For KREC, the model showed that lowering the cut‐off from <6 to <4 copies/punch reduced abnormal results from 121 to 44 (a 64% reduction, *p* < .0001) without missing any cases of agammaglobulinemia. The ROC analysis was consistent with this finding, demonstrating 100% relative sensitivity improved specificity compared with the <6 cut‐off (Figure S3B).

### 3.3 Impact of risk stratification

**FIGURE 1:**
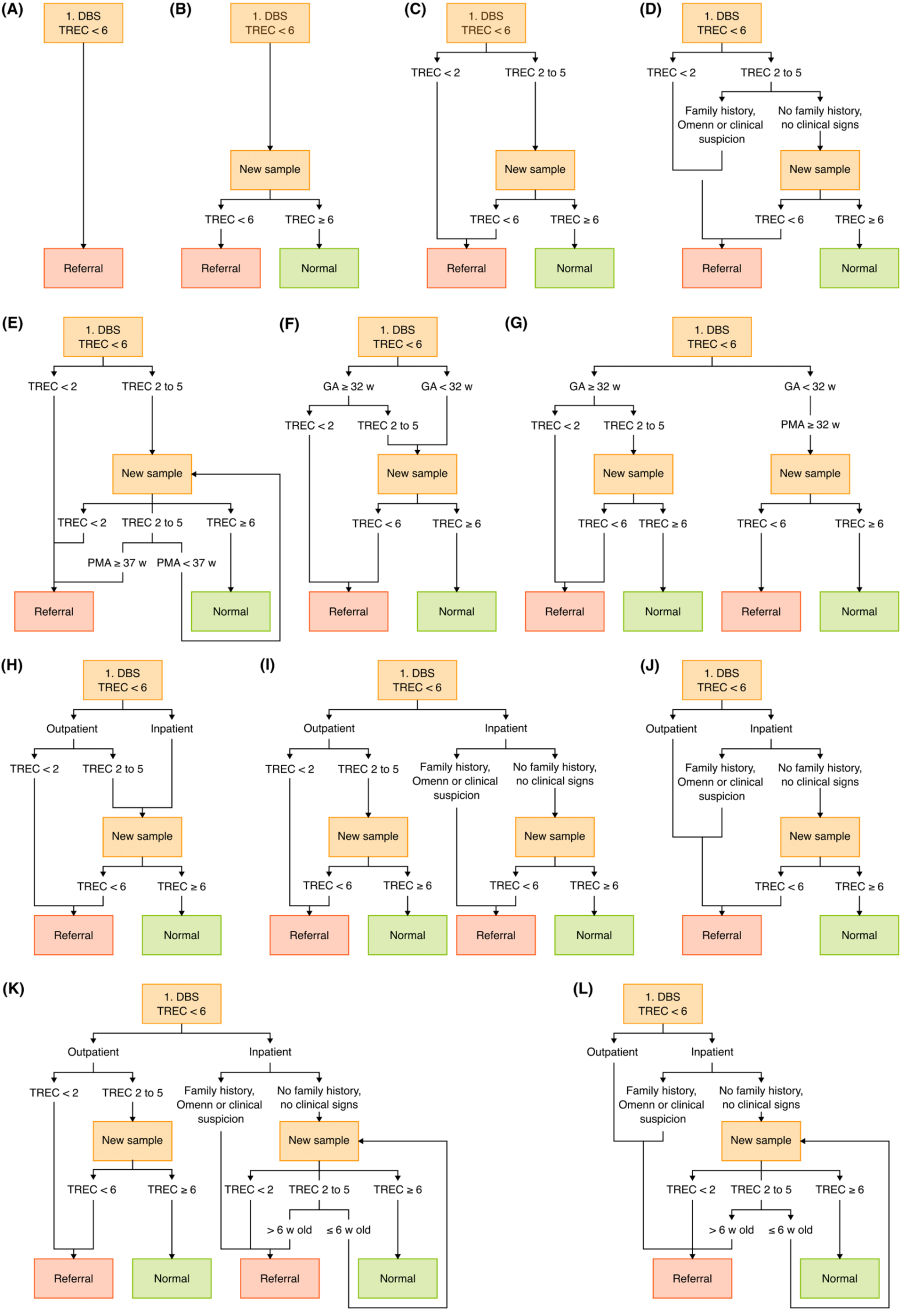
Screening algorithms for abnormal T‐cell receptor excision circles (TREC). DBS, dried blood spot; GA, gestational age; PMA, postmenstrual age; w, week.

**FIGURE 2:**
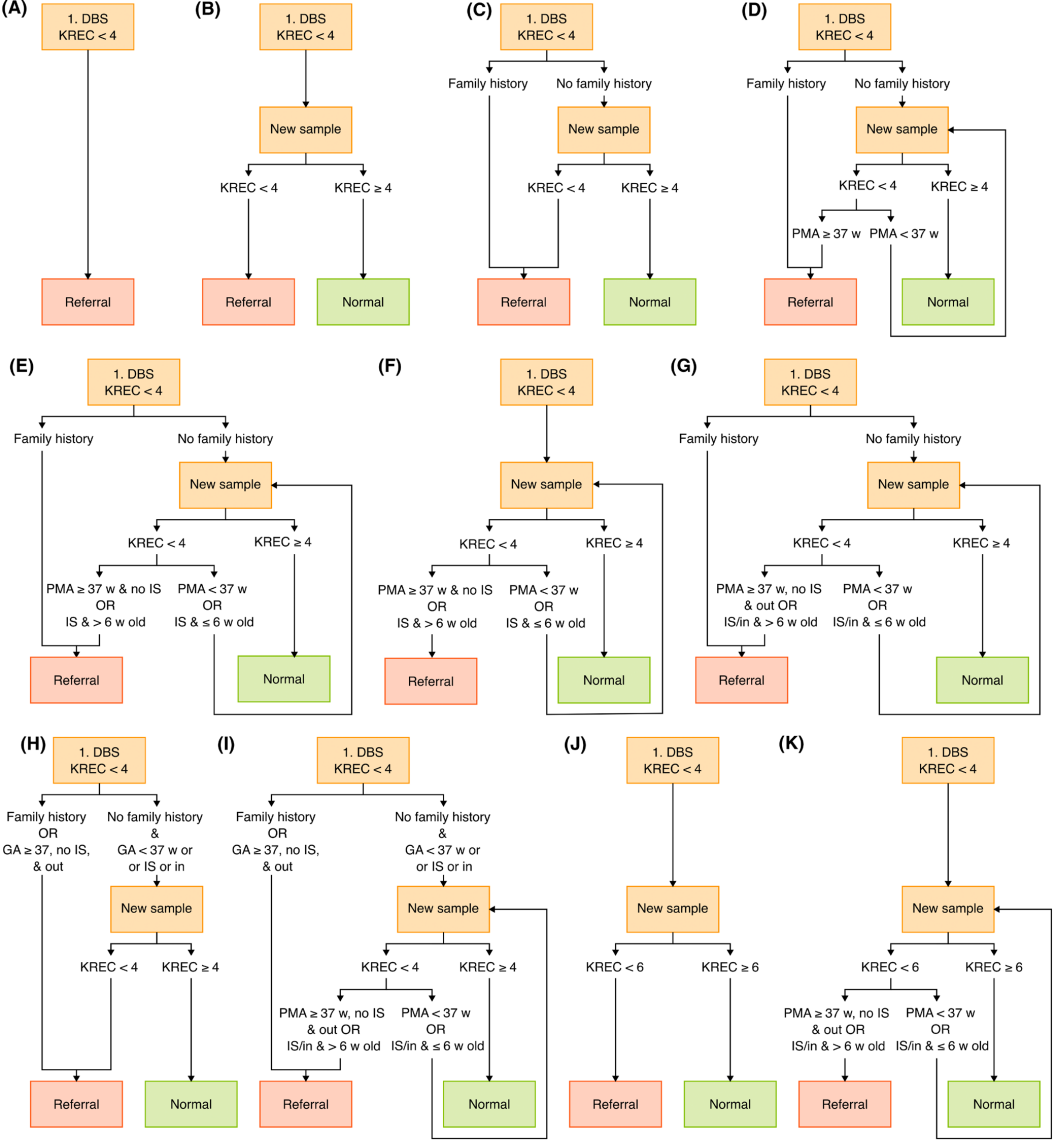
Screening algorithms for abnormal kappa‐deleting recombination excision circles (KREC). DBS, dried blood spot, GA, gestational age; in, inpatient; IS, maternal immunosuppression; out, outpatient; PMA, post‐menstrual age; w, week.

Risk‐stratification could be modeled using data from 116 newborns for TREC and 138 for KREC (newborns with initial TREC <6 and/or KREC <4 and a complete dataset, details on the excluded newborns can be found in Table S3). The developed algorithms can be found in Figures 1 and 2. Algorithms TREC A and KREC A provided a baseline with all individuals with abnormal results being referred.

Risk‐stratified modeling for TREC demonstrated that requiring a second DBS for all abnormal cases (TREC B) reduced referrals by 57% compared with baseline (*p* < .0001) but delayed diagnosis in all cases of SCID and congenital athymia. Introducing an “urgency” classification based on exact TREC values (TREC C) avoided these delays and still reduced referrals by 39% to baseline (*p* < .0001). Adding family history and clinical signs to the urgent referral criteria (TREC D) yielded identical outcomes to TREC C. The greatest reduction in referrals (61% compared with baseline, *p* < .0001) was achieved in TREC E, which combined urgency‐based stratification with repeated sampling in preterm infants until they reached a PMA of 37 weeks. However, this algorithm missed almost half of the moderate TCL cases (47%). Other models combining urgency criteria with GA thresholds, inpatient status, family history, clinical signs or multiple repeat samples (TREC F–L) achieved varying balances between reducing referrals, avoiding missed cases, and minimizing delays. An overview of the applied criteria is provided in Table 1, the resulting outcomes in Table 2 and detailed statistical comparisons of the resulting referrals in Table S4.

**TABLE 1:** Measures applied in alternative TREC algorithms.

Measure	TREC algorithm											
	A	B	C	D	E	F	G	H	I	J	K	L
*General approach*												
Direct referrals in all												
New DBS sample for all												
*Criterium of stratification or additional measure*												
Urgency												
Family history or clinical signs												
Prematurity												
Delaying repeat DBS sample												
In‐versus outpatient												
Multiple repeat DBS samples												

*Note:* Measures applied in the respective algorithm are shaded blue.

Abbreviations: DBS, dried blood spot; TREC, T‐cell receptor excision circles.

**TABLE 2:** Comparison of modeled screening algorithms for TREC: outcome measures, detected and missed cases.

	TREC algorithms											
	A	B	C	D	E	F	G	H	I	J	K	L
*Outcomes*												
Referrals	116	50	71	71	45	67	56	59	59	78	52	71
Total new samples^a^	0	116	64	64	90	72	72	92	89	64	100	75
*Detected cases*												
SCID	11	11	11	11	11	11	11	11	11	11	11	11
Athymia	2	2	2	2	2	2	2	2	2	2	2	2
TCL	32	19	20	20	17	20	20	19	19	27	17	25
‐actionable TCL	24	15	16	16	15	16	16	15	15	23	15	21
‐non‐actionable TCL	8	4	4	4	2	4	4	4	4	6	2	4
*Missed cases*												
TCL	0	13	12	12	15	12	12	13	13	5	15	7
‐actionable												
22q11.2DS		4	4	4	4	4	4	4	4	2	4	2
Idiopathic TCL		3	3	3	3	3	3	3	3		3	
Unclear TCL		1	1	1	1	1	1	1	1		1	
‐non‐actionable												
Idiopathic TCL		2	2	2	2	2	2	2	2	2	2	2
Trisomy 21					1						1	1
Unclear syndromic disease with TCL		1			2			1	1	1	2	2
VUS in PU.1		1	1	1	1	1	1	1	1		1	
Unclear TCL		1	1	1	1	1	1	1	1		1	
*Delayed diagnosis*												
SCID	0	11	0	0	0	0	0	3	2	2	2	2
Athymia	0	2	0	0	0	0	0	1	0	0	0	0
Outpatients	0	49	25	25	34	25	25	25	25	0	25	0

Abbreviations: 22q11.2DS, 22q.11.2 deletion syndrome, AT, ataxia‐telangiectasia; KREC, kappa‐deleting recombination excision circles; SCID, severe combined immunodeficiency; TCL, T‐cell lymphopenia; TREC, T‐cell receptor excision circle; VUS, variant of uncertain significance.

^a^ Number of all additional dried blood spot samples obtained as a result of applying the algorithm.

For KREC, obtaining a second DBS in all abnormal cases (KREC B) reduced referrals by 71% (*p* < .0001) but missed two cases of λ5 deficiency. The features used for stratification in the alternative KREC algorithms are summarized in Table 3. Additional stratification based on family history (KREC C) did not recover the missed cases of λ5 deficiency but reduced delays in other confirmed agammaglobulinemia patients. Incorporating repeated sampling in preterm infants until reaching term (KREC D) further reduced referrals (78% compared to baseline, *p* < .0001). Adding maternal immunosuppression as a stratification factor (KREC E) and allowing multiple DBS repeats in both preterm infants and those with in utero exposure to immunosuppressive medication reduced referrals by 89% compared to baseline (*p* < .0001). Including inpatient status as an additional factor to the prior (KREC G) produced the lowest referral rate overall, reducing referrals by 91% compared to baseline (*p* < .0001), while identifying all agammaglobulinemia cases except the two with λ5 deficiency. Algorithms designed to detect all λ5 deficiency cases, such as increasing KREC cut‐offs for repeat testing (KREC J), achieved complete detection but at the cost of substantially higher referral rates. Outcomes of the modeled KREC algorithms are presented in Table 4. A detailed comparison of KREC algorithms is provided in Table S5.

**TABLE 3:** Features used for stratification in alternative KREC algorithms.

Measure	KREC algorithm										
	A	B	C	D	E	F	G	H	I	J	K
*General approach*											
New DBS sample for all											
*Criterium of stratification or additional measure*											
Family history or clinical signs											
Prematurity											
Maternal immunosuppression											
Multiple repeat DBS samples											
In‐ vs. outpatient											
Increased cut‐offs for new DBS											

*Note:* Measures applied in the respective algorithm are shaded blue.

Abbreviations: DBS, dried blood spot; KREC, kappa‐deleting recombination excision circles.

**TABLE 4:** Comparison of modeled screening algorithms for KREC: outcome measures, detected and missed cases.

	KREC algorithms										
	A	B	C	D	E	F	G	H	I	J	K
*Outcome measures*											
Referrals	138	40	40	30	15	15	13	81	54	54	21
Total new samples^a^	0	138	136	151	167	169	169	85	54	54	21–25^b^
*Detected cases*											
Agammaglobulinemia	11	9	9	9	9	9	9	10	10	11	11
Moderate TCL (AT)	1	1	1	1	1	1	1	1	1	1	1
*Missed cases*											
Agammaglobulinemia (λ5 deficiency)	0	2	2	2	2	2	2	1	1	0	0

Abbreviations: AT, ataxia‐telangiectasia; KREC, kappa‐deleting recombination excision circles; TCL, T‐cell lymphopenia.

^a^ Number of all additional dried blood spot samples obtained as a result of applying the algorithm.

^b^ Exact number could not be calculated due to the lack of a 3rd dried blood spot sample in 4 newborns.

## 4 DISCUSSION

This study modeled the impact of alternative NBS algorithms for TREC‐ and KREC‐based detection of severe T‐ and B‐cell lymphopenia using a comprehensive, prospectively collected dataset from 6 years of the Swiss NBS program. The results demonstrate that incorporating readily available or easily obtainable clinical information such as exact TREC value, GA at birth, PMA at sampling, inpatient vs. outpatient status, family history, and maternal immunosuppression can substantially reduce unnecessary referrals without compromising detection of the main target conditions. For TREC, the most efficient algorithm reduced referrals by 61% compared with the baseline approach, while for KREC, the combination of prematurity, maternal immunosuppression, and inpatient status yielded a 91% reduction in referrals.

In the TREC models, the lowest referral rate was achieved by directly referring newborns with almost unmeasurable TREC values and subjecting all others to repeat sampling, with additional repeat testing in preterm infants until they reached a PMA of 37 weeks. This algorithm (TREC E) ensured timely referral of all SCID and congenital athymia cases but missed nearly half of the newborns with non‐SCID, non‐congenital athymia TCL. The benefit of detecting these milder forms of TCL through NBS remains debated; however, previous work, including our own, has shown that a substantial proportion of such cases have actionable findings.^20^, ^36^, ^37^, ^38^, ^39^ While TREC E is straightforward to implement, it prolongs the period of uncertainty in more than two‐thirds of outpatients who ultimately require referral, a factor that may increase parental anxiety.^40^

Where resources allow, early communication with healthcare providers and families, more sophisticated strategies can be considered. For example, an approach combining urgency of TREC values with inpatient status, family history, and syndromic features (TREC K) achieved similar referral reductions to TREC E but shortened delays for some outpatients, albeit at a cost of delayed referral in 20% of SCID patients. Direct referral of all outpatients, as in the current Swiss practice (TREC L), further reduces uncertainty for families but increases referral rates compared with TREC K.

For KREC, the largest reduction in referrals was achieved by applying repeated sampling in newborns with factors associated with transiently abnormal results^14^: prematurity, in utero exposure to maternal immunosuppression, and inpatient status. This approach (KREC G) reduced referrals to less than 10% of abnormal cases, while retaining all confirmed agammaglobulinemia patients except some of those with λ5 deficiency. Although maternal immunosuppression is not routinely recorded in many programs, it could be captured through simple modifications to NBS cards. One limitation of the multistep KREC algorithms is the extended periods of uncertainty, which could be mitigated through timely counseling. Detecting all cases of agammaglobulinemia related to variants in *IGLL1* (λ5 deficiency) was only possible by increasing the KREC cut‐off for repeat sampling, a change that substantially increased referral rates. The long‐term benefit of early diagnosis in λ5 deficiency is not yet well defined, and its inclusion as a primary NBS target remains debatable.^41^

This is the first study to model stratified algorithms for KREC and one of the most detailed evaluations of TREC algorithm optimization to date. Its strengths include the use of a large, high‐quality, real‐life dataset and the ability to model multiple clinically relevant stratification variables. Limitations include the exclusion of some cases due to incomplete data and the restriction of modeling to variables available in the registry. Moreover, local differences in resources, follow‐up pathways, and parental support mean that no single algorithm will suit all NBS programs. In Switzerland, we have adopted TREC algorithm L, allowing direct referral of outpatients and those with features suggestive of SCID or congenital athymia, while subjecting inpatients to one or more repeat samples before referring. This approach helps control the number of referrals while avoiding delays in infants at high risk of SCID and reducing parental stress among outpatients. For KREC, we have implemented algorithm G, which similarly permits direct referral of infants with a positive family history, while preterm infants, inpatients, and newborns with in utero exposure to maternal immunosuppression undergo repeated testing to minimize unnecessary referrals. In settings where obtaining these additional clinical details is feasible, we would recommend this approach to others as well.

In conclusion, stratifying newborns with abnormal TREC or KREC results based on simple, readily obtainable clinical information markedly improves the specificity of NBS for severe T‐ and B‐cell lymphopenia. Such approaches can reduce the burden on healthcare systems and families without compromising the detection of primary target diseases, and they provide a practical, adaptable framework for optimizing NBS algorithms in diverse healthcare settings.

## AUTHOR CONTRIBUTIONS

**Susanna Sluka:** Writing – review and editing; data curation. **Seraina Prader:** Writing – review and editing; data curation. **Maarja Soomann:** Conceptualization; investigation; funding acquisition; writing – original draft; methodology; visualization; writing – review and editing; formal analysis; project administration; data curation. **Jana Pachlopnik Schmid:** Data curation; funding acquisition; writing – review and editing; supervision. **Johannes Trück:** Supervision; data curation; funding acquisition; writing – review and editing; writing – original draft.

## FUNDING INFORMATION

M.S. was funded by Filling the Gap, a career development program of the University of Zurich, Switzerland. JPS received funding from the Swiss National Science Foundation (320030_205097) and ITINERARE, a University Research Priority Program of the University of Zurich, Switzerland. The funding agencies did not influence the study design, the collection, analysis, or interpretation of data, the writing of the report, nor the decision to submit the paper for publication.

## CONFLICT OF INTEREST STATEMENT

None of the authors have any conflicts of interest related to this work.

## ETHICS STATEMENT

This study was performed in line with the principles of the Declaration of Helsinki. Approval was granted permission by the Cantonal Ethics Commission of Zurich (2022‐01029).

## Supporting information


**Figure S1:** Distribution of patients with a final diagnosis of an inborn error of immunity based on their gestational age. SCID, Severe combined immunodeficiency; TCL, T‐cell lymphopenia.
**Figure S2:** Effect of reducing cut‐offs. Each dot represents a newborn, color‐coded by category of final findings. Solid lines represent the lower (TREC <6, KREC <4) and dashed lines the higher cut‐offs (TREC <10, KREC <6 copies/punch). Using lower cut‐offs, those on the shaded area would have been classified as normal leading to missing the TCL cases labeled red. iTCL, idiopathic T‐cell lymphopenia; SCID, severe combined immunodeficiency; TCL, T‐cell lymphopenia; UTL, unclear T‐cell lymphopenia (no genetic testing).
**Figure S3:** Receiver Operating Characteristic (ROC) curves for TREC (A) and KREC (B). Each dot represents a specific cut‐off.
**Table S1:** Packages used for data analysis in R version 4.2.2.
**Table S2:** Final diagnoses in patients identified to have a non‐secondary T‐ or B‐cell lymphopenia.
**Table S3:** Excluded patients.
**Table S4:** Comparison of referral rates in modeled algorithms TREC A to L.
**Table S5:** Comparison of referral rates in modeled algorithms KREC A to K.

## Data Availability

The data that support the findings of this study are available on request from the corresponding author. The data are not publicly available due to privacy or ethical restrictions.
