# Supplementary material for: Optimizing TREC‐ and KREC‐based newborn screening: Risk‐stratified algorithms significantly reduce referrals
Source: Pediatr Allergy Immunol. 2026 Jul 8;37(7):e70412. doi: 10.1111/pai.70412 (PMC13344459; doi:10.1111/pai.70412)
Supplement: Supplementary file 1 — Figure S1: Distribution of patients with a final diagnosis of an inborn error of immunity based on their gestational age. SCID, Severe combined immunodeficiency; TCL, T‐cell lymphopenia. Figure S2: Effect of reducing cut‐offs. Each dot represents a newborn, color‐coded by category of final findings. Solid lines represent the lower (TREC <6, KREC <4) and dashed lines the higher cut‐offs (TREC <10, KREC <6 copies/punch). Using lower cut‐offs, those on the shaded area would have been classified as normal leading to missing the TCL cases labeled red. iTCL, idiopathic T‐cell lymphopenia; SCID, severe combined immunodeficiency; TCL, T‐cell lymphopenia; UTL, unclear T‐cell lymphopenia (no genetic testing). Figure S3: Receiver Operating Characteristic (ROC) curves for TREC (A) and KREC (B). Each dot represents a specific cut‐off. Table S1: Packages used for data analysis in R version 4.2.2. Table S2: Final diagnoses in patients identified to have a non‐secondary T‐ or B‐cell lymphopenia. Table S3: Excluded patients. Table S4: Comparison of referral rates in modeled algorithms TREC A to L. Table S5: Comparison of referral rates in modeled algorithms KREC A to K. [file PAI-37-e70412-s001.pdf]

Supporting Information to

Optimizing TREC- and KREC-based Newborn Screening: Risk-Stratified Algorithms Significantly Reduce Referrals

Soomann *et al* 2026

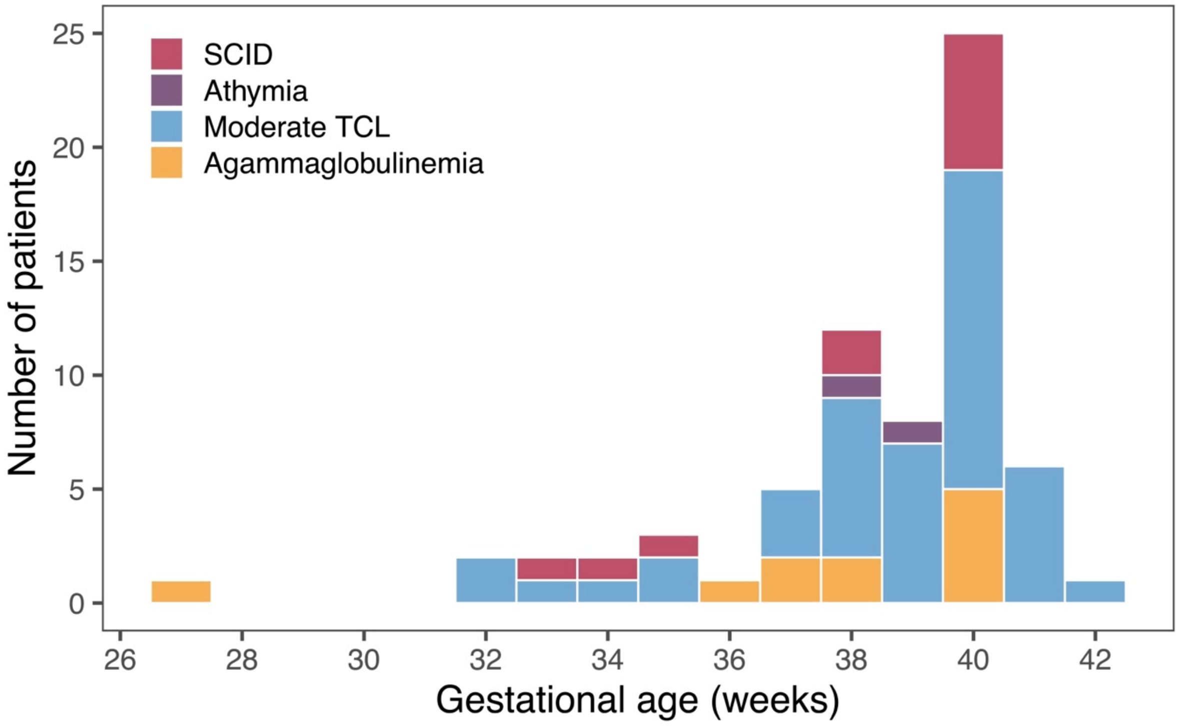

Figure S1. Distribution of patients with a final diagnosis of an inborn error of immunity based on their gestational age. *SCID, Severe combined immunodeficiency; TCL, T-cell lymphopenia.*

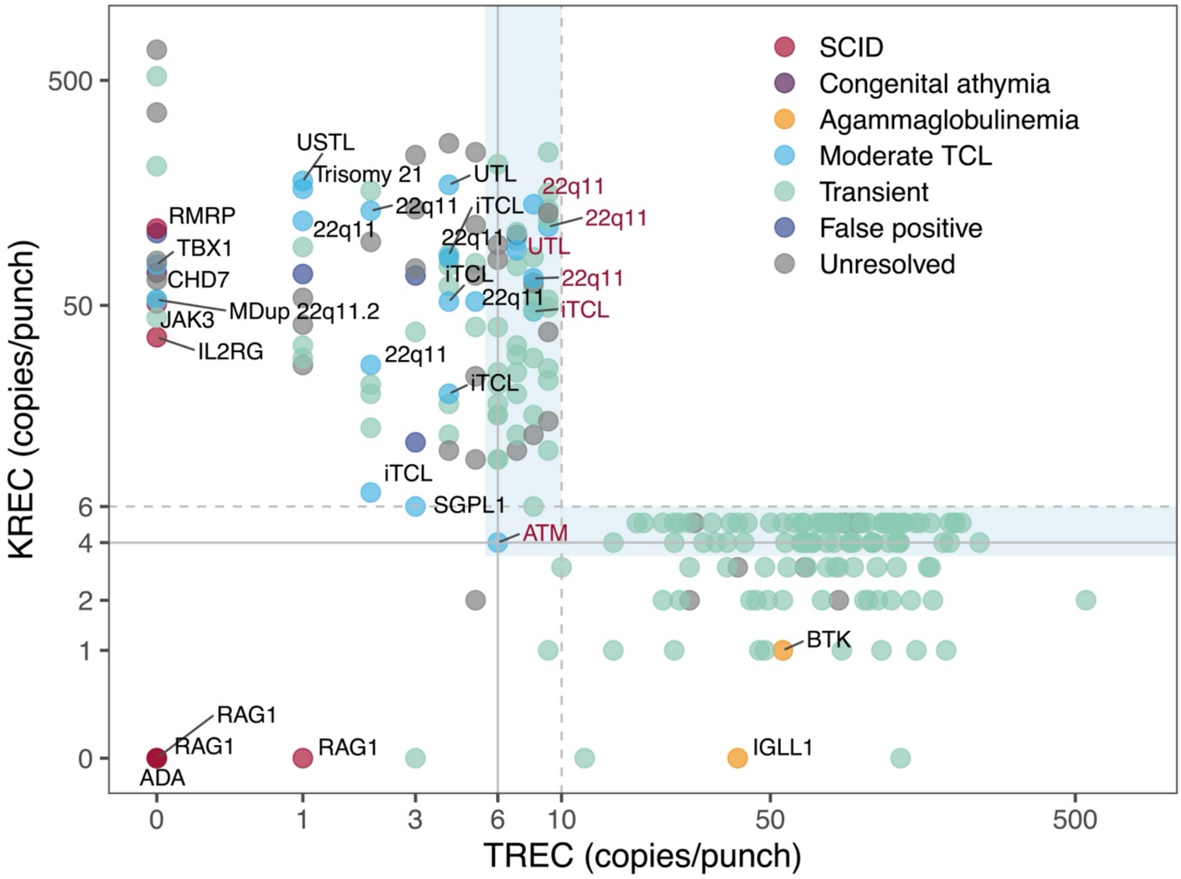

Figure S2. Effect of reducing cut-offs. Each dot represents a newborn, color-coded by category of final findings. Solid lines represent the lower (TREC <6, KREC <4) and dashed lines the higher cut-offs (TREC <10, KREC <6 copies/punch). Using lower cut-offs, those on the shaded area would have been classified as normal leading to missing the TCL cases labelled red. *iTCL, idiopathic T-cell lymphopenia; SCID, severe combined immunodeficiency; TCL, T-cell lymphopenia; UTL, unclear T-cell lymphopenia (no genetic testing)*

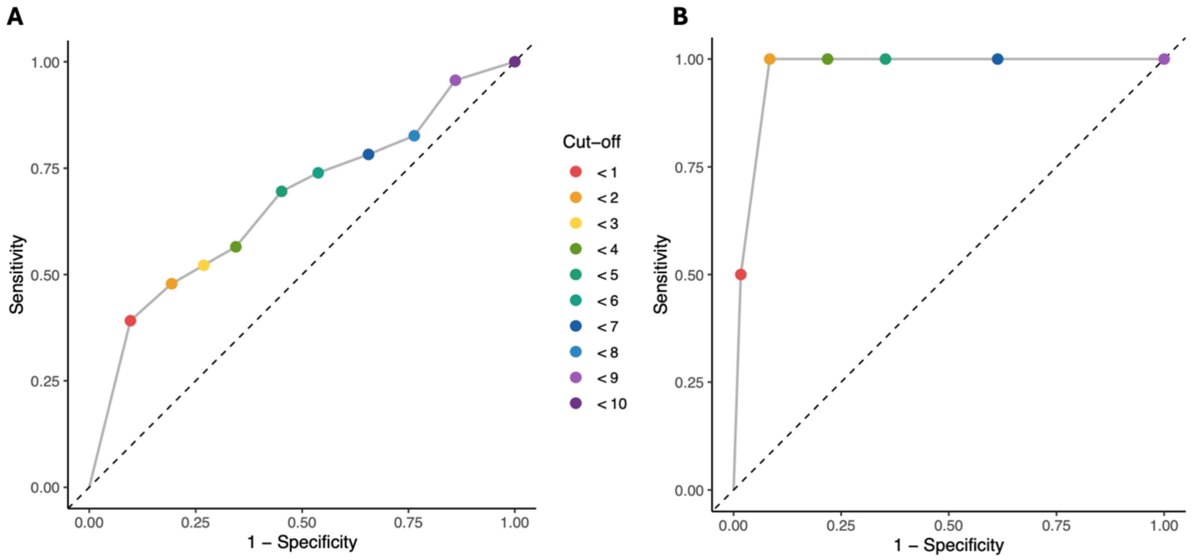

Figure S3. Receiver Operating Characteristic (ROC) curves for TREC (A) and KREC (B). Each dot represents a specific cut-off.

Table S1. Packages used for data analysis in R version 4.2.2

Type	Packages
Base packages	stats, graphics, grDevices, utils, datasets, methods, base
Additional packages	ggrepel 0.9.3, knitr 1.42, ggthemes 4.2.4, readxl 1.4.2, stringr 1.5.1, dplyr 1.1.0, purrr 1.0.1, tidyr 1.3.0, tibble 3.1.8, tidyverse 2.0.0, Hmisc 4.8.0, ggplot2 3.4.1, RORC 1.0.12

Table S2. Final diagnoses in patients identified to have a non-secondary T- or B-cell lymphopenia

Group	Gene/Findings	Number
Abnormal TREC		***56***
**Severe combined immunodeficiency**		**11**
T −B −		
ADA deficiency	*ADA*	2
RAG deficiency	*RAG1*	3
T −B+		
γc deficiency	*IL2RG*	2
IL7Rα deficiency	*IL7RA*	1
JAK3 deficiency	*JAK3*	1
Cartilage hair hypoplasia	*RMRP*	1
Undefined	none	1
**Congenital athymia**		**2**
22q11.2 deletion syndrome	MD22q11.2	1
CHARGE syndrome	*CHD7*	1
**Moderate T-cell lymphopenia**		**43**
Combined immunodeficiency with associated or syndromic features		
22q11.2 deletion syndrome	MD22q11.2	15
Ataxia-telangiectasia	*ATM*	1
FOXN1 haploinsufficiency	*FOXN1*	1
Microduplication syndrome 22q11.2	MDup22q11.2	1
SGPL1 deficiency	*SGPL1*	1
Other syndromic diseases		
Trisomy 21	Trisomy 21	1
Known syndromes, novel association with TCL		
	ZMIZ1	1
	ANKRD11	1
Unclear syndromic disease despite genetic testing		2
Other		
Idiopathic T-cell lymphopenia	none	11
Variants of unknown significance in inborn errors of immunity genes		
	TBX1	1
	LCK	1
	PU.1	1
No genetic testing performed		5
Abnormal KREC		***12***
**Agammaglobulinemia**		**11**
X-linked agammaglobulinemia	*BTK*	2
λ5 deficiency	*IGLL1*	9
**Moderate T-cell lymphopenia**		**1**
Ataxia-telangiectasia	*ATM*	1

TCL, T-cell lymphopenia

Table S3. Excluded patients

Modelling cohort	Number
**TREC**	**76**
**Reason for exclusion**	**76**
initial TREC levels between 6 and 9 copies/punch	53
lack of a second DBS sample	23
deceased before a new sample could be obtained	*14*
lost to follow-up	*5*
referred without obtaining additional samples	*4*
**Excluded IEI patients**	**9**
22q11.2 deletion syndrome	4
ataxia telangiectasia	1
SGPL1 deficiency	1
variant of unknown significance in TBX1	1
idiopathic T-cell lymphopenia	1
unclear syndromic disease	1
no genetic testing	2
**KREC**	**84**
**Reason for exclusion**	**84**
initial KREC between 4 and 6 copies/punch	77
lack of a second DBS sample	7
lost to follow-up	*3*
deceased before a new sample could be obtained	*1*
referred without obtaining additional samples	*3*
**Excluded IEI patients**	**0**

Table S4. Comparison of referral rates in modelled algorithms TREC A to L

	A	B	C	D	E	F	G	H	I	J	K	L
**A**	–	-57% (p<0.0001)	-39% (p<0.0001)	-39% (p<0.0001)	-61% (p<0.0001)	-42% (p<0.0001)	-52% (p<0.0001)	-49% (p<0.0001)	-49% (p<0.0001)	-33% (p<0.0001)	-55% (p<0.0001)	-39% (p<0.0001)
**B**	+132% (p<0.0001)	–	+42% (p=0.009)	+42% (p=0.009)	-10% (p=0.59)	+34% (p=0.04)	+12% (p=0.51)	+18% (p=0.29)	+18% (p=0.29)	+56% (p=0.0004)	+4% (p=0.89)	+42% (p=0.009)
**C**	+63% (p<0.0001)	-30% (p=0.009)	–	+0% (p=1)	-37% (p=0.001)	-6% (p=0.69)	-21% (p=0.07)	-17% (p=0.15)	-17% (p=0.15)	+10% (p=0.41)	-27% (p=0.02)	+0% (p=1)
**D**	+63% (p<0.0001)	-30% (p=0.009)	+0% (p=1)	–	-37% (p=0.001)	-6% (p=0.69)	-21% (p=0.07)	-17% (p=0.15)	-17% (p=0.15)	+10% (p=0.41)	-27% (p=0.02)	+0% (p=1)
**E**	+158% (p<0.0001)	+11% (p=0.59)	+58% (p=0.001)	+58% (p=0.001)	–	+49% (p=0.006)	+24% (p=0.19)	+31% (p=0.09)	+31% (p=0.09)	+73% (p<0.0001)	+16% (p=0.42)	+58% (p=0.001)
**F**	+73% (p<0.0001)	-25% (p=0.04)	+6% (p=0.69)	+6% (p=0.69)	-33% (p=0.006)	–	-16% (p=0.19)	-12% (p=0.36)	-12% (p=0.36)	+16% (p=0.18)	-22% (p=0.07)	+6% (p=0.69)
**G**	+107% (p<0.0001)	-11% (p=0.51)	+27% (p=0.07)	+27% (p=0.07)	-20% (p=0.19)	+20% (p=0.19)	–	+5% (p=0.79)	+5% (p=0.79)	+39% (p=0.005)	-7% (p=0.69)	+27% (p=0.07)
**H**	+97% (p<0.0001)	-15% (p=0.29)	+20% (p=0.15)	+20% (p=0.15)	-24% (p=0.09)	+14% (p=0.36)	-5% (p=0.79)	–	+0% (p=1)	+32% (p=0.02)	-12% (p=0.43)	+20% (p=0.15)
**I**	+97% (p<0.0001)	-15% (p=0.29)	+20% (p=0.15)	+20% (p=0.15)	-24% (p=0.09)	+14% (p=0.36)	-5% (p=0.79)	+0% (p=1)	–	+32% (p=0.02)	-12% (p=0.43)	+20% (p=0.15)
**J**	+49% (p<0.0001)	-36% (p=0.0004)	-9% (p=0.41)	-9% (p=0.41)	-42% (p<0.0001)	-14% (p=0.18)	-28% (p=0.005)	-24% (p=0.02)	-24% (p=0.02)	–	-33% (p=0.001)	-9% (p=0.41)
**K**	+123% (p<0.0001)	-4% (p=0.89)	+37% (p=0.02)	+37% (p=0.02)	-13% (p=0.42)	+29% (p=0.07)	+8% (p=0.69)	+13% (p=0.43)	+13% (p=0.43)	+50% (p=0.001)	–	+37% (p=0.02)
**L**	+63% (p<0.0001)	-30% (p=0.009)	+0% (p=1)	+0% (p=1)	-37% (p=0.001)	-6% (p=0.69)	-21% (p=0.07)	-17% (p=0.15)	-17% (p=0.15)	+10% (p=0.41)	-27% (p=0.02)	–

Table S5. Comparison of referral rates in modelled algorithms KREC A to K

	A	B	C	D	E	F	G	H	I	J	K
**A**	–	-71% (p<0.0001)	-71% (p<0.0001)	-78% (p<0.0001)	-89% (p<0.0001)	-89% (p<0.0001)	-91% (p<0.0001)	-41% (p<0.0001)	-61% (p<0.0001)	-61% (p<0.0001)	-85% (p<0.0001)
**B**	+245% (p<0.0001)	–	+0% (p=1)	-25% (p=0.21)	-63% (p=0.0003)	-63% (p=0.0003)	-68% (p<0.0001)	+103% (p<0.0001)	+35% (p=0.10)	+35% (p=0.10)	-48% (p=0.009)
**C**	+245% (p<0.0001)	+0% (p=1)	–	-25% (p=0.21)	-63% (p=0.0003)	-63% (p=0.0003)	-68% (p<0.0001)	+103% (p<0.0001)	+35% (p=0.10)	+35% (p=0.10)	-48% (p=0.009)
**D**	+360% (p<0.0001)	+33% (p=0.21)	+33% (p=0.21)	–	-50% (p=0.023)	-50% (p=0.023)	-57% (p=0.0079)	+170% (p<0.0001)	+80% (p=0.003)	+80% (p=0.003)	-30% (p=0.21)
**E**	+820% (p<0.0001)	+167% (p=0.0003)	+167% (p=0.0003)	+100% (p=0.023)	–	+0% (p=1)	-13% (p=0.84)	+440% (p<0.0001)	+260% (p<0.0001)	+260% (p<0.0001)	+40% (p=0.37)
**F**	+820% (p<0.0001)	+167% (p=0.0003)	+167% (p=0.0003)	+100% (p=0.023)	+0% (p=1)	–	-13% (p=0.84)	+440% (p<0.0001)	+260% (p<0.0001)	+260% (p<0.0001)	+40% (p=0.37)
**G**	+962% (p<0.0001)	+208% (p<0.0001)	+208% (p<0.0001)	+131% (p=0.008)	+15% (p=0.84)	+15% (p=0.84)	–	+523% (p<0.0001)	+315% (p<0.0001)	+315% (p<0.0001)	+61% (p=0.2)
**H**	+70% (p<0.0001)	-51% (p<0.0001)	-51% (p<0.0001)	-63% (p<0.0001)	-82% (p<0.0001)	-82% (p<0.0001)	-84% (p<0.0001)	–	-33% (p=0.002)	-33% (p=0.002)	-74% (p<0.0001)
**I**	+156% (p<0.0001)	-26% (p=0.10)	-26% (p=0.10)	-44% (p=0.003)	-72% (p<0.0001)	-72% (p<0.0001)	-76% (p<0.0001)	+50% (p=0.002)	–	+0% (p=1)	-61% (p<0.0001)
**J**	+156% (p<0.0001)	-26% (p=0.10)	-26% (p=0.10)	-44% (p=0.003)	-72% (p<0.0001)	-72% (p<0.0001)	-76% (p<0.0001)	+50% (p=0.002)	+0% (p=1)	–	-61% (p<0.0001)
**K**	+557% (p<0.0001)	+91% (p=0.009)	+91% (p=0.009)	+43% (p=0.21)	-29% (p=0.37)	-29% (p=0.37)	-38% (p=0.2)	+286% (p<0.0001)	+157% (p<0.0001)	+157% (p<0.0001)	–
